# Supplementary material for: NSD3 protein methylation and stabilization transforms human ES cells into variant state
Source: Life Sci Alliance. 2024 Dec 31;8(3):e202402871. doi: 10.26508/lsa.202402871 (PMC11707394; doi:10.26508/lsa.202402871)
Supplement: Supplementary file 3 [file LSA-2024-02871_Supplemental_Data_1.docx]

**NSD3 protein methylation and stabilization transforms human ES cells into variant state**

Vignesh K Krishnamoorthy^1,2^, Fariha Hamdani^1^, Pooja Shukla^1^, Radhika Rao Arsala^3^, Shaikh Anaitullah^3,^ Kriti Kestur Biligiri^1,2^, Rajashekar Varma Kadumuri^4^, Purushotham Reddy Pothula^5^, Sreenivas Chavali^4^ and Shravanti Rampalli ^1,2 *^

**Sequencing results of NSD3-Long Wt.**

CCCCATTCTCATTTTCCACTGGAGACATGTTACACTTCTGCAAATCCAGGCTCCCTTTGTGCATCGTAATGGAAGCTGGTAAGGA**TTT**CCTTGCTGCCGCAGTTTTCCAGGCTATTTTAACAGGCGGTGGCTCTTCCTCTTCCGCACTTGTGTGCCGCCTCTGGCTATGTCTCCGAATTTCAGTACTTGAGAGTGAGGAGGCCACCTCCCCTGCATTGGTCTGTTCTGGCTGAGTATTCAGCACAGATCTTGGTCGTCGGGTTTTTTTAACTTCGGTTTTGGAGGCAACACTCTTTTTTGCTTGGGATAAAGCCTCTTCAGGCTGTTTATCAATGTAAATAAAAGTATACTGTTCTATTCTTTCTTCTCGAGTCATTTTCAATGCTTTCTCTGCATGGGCAATGCCAATATCCCACTGAGCACGTTCTCTCTGAGGTCGGGGTTTCCGAATCTTTTGTTTCTCAGAGTGATTGCTGGCTTGTTTGGTTGCCTCAGCCAGTAATTCTTCATACTGTTTATGACCTTTATACTCTCGTACCCGTTTTTCATGAACCCACGCCCTCTCTGGCTGGTTGCTAAAAAACTGGACATGATATTCTCGGGCACCTCTTGTGTTAATTTTAGTATGAACCTCAAGCTGGGGATCACTTGAAACCATACAAGGCCACCAAGGATAGGTTCCCACCTTGGACCACACAAGATCGCCAACCTGAAACTTAACACCAGTGGACACTTCCGTTGTTGGAACAGAAGATAGTATTGGCTGAACTGGGGGCTTCCTCTTTTAGTACTGGTTCTTCCCTTGGTTTTTCTGATACAGTGTCAACCCTCTCATTTGGTCTATTTTGTTCCTCTGGTTCTAATTTGGGGGATTTTGTGTGACTTGCGCTCTTCAGATCTTGATGAGTCATGCTTGTTGCTTTTTTTCCTCTTTTCTTTTCTGCTTTCATGCTTTGATTTCGTGTGCTCACTTGCCTGTACTTCATTTAAAAGGTCTCCACAAAGGGAAGACTCAAACAATTCCCTGCCATTCTGGATAGTTTTGGTTATTTTAAGTTTAATTTCAGTTGAGCAAGTCTTCTTGCATCCACAGTTTGTGGTACCGAAGAGGAGGTGGTGCTTGTGAAGGGAC

**Sequencing results of K477R Mutant of NSD3-Long**

AAAAACCTTGTTTTCATTTTAACAACTGGAGACATGTTACACTTCTGCAAATCCAGGCTCCCTTTGTGCATCGTAATGGAAGCTGGTAAGGA**TCT**CCTTGCTGCCGCAGTTTTCCAGGCTATTTTAACAGGCGGTGGCTCTTCCTCTTCCGCACTTGTGTGCCGCCTCTGGCTATGTCTCCGAATTTCAGTACTTGAGAGTGAGGAGGCCACCTCCCCTGCATTGGTCTGTTCTGGCTGAGTATTCAGCACAGATCTTGGTCGTCGGGTTTTTTTAACTTCGGTTTTGGAGGCAACACTCTTTTTTGCTTGGGATAAAGCCTCTTCAGGCTGTTTATCAATGTAAATAAAAGTATACTGTTCTATTCTTTCTTCTCGAGTCATTTTCAATGCTTTCTCTGCATGGGCAATGCCAATATCCCACTGAGCACGTTCTCTCTGAGGTCGGGGTTTCCGAATCTTTTGTTTCTCAGAGTGATTGCTGGCTTGTTTGGTTGCCTCAGCCAGTAATTCTTCATACTGTTTATGACCTTTATACTCTCGTACCCGTTTTTCATGAACCCACGCCCTCTCTGGCTGGTTGCTAAAAAACTGGACATGATATTCTCGGGCACCTCTTGTGTTAATTTTAGTATGAACCTCAAGCTGGGGATCACTTGAAACCATACAAGGCCACCAAGGATAGGTTCCCACCTTGGACCACACAAGATCGCCAACCTGAAACTTAACACCAGTGGACACTTCCGTTGTTGGAACAGAAGATAGTATTGGCTGAACTGGGGCTTCCTCTTTTAGTACTGGTTCTTCCCTTGGTTTTTCTGATACAGTGTCAACCCTCTCATTTGGTCTATTTTGTTCCTCTGGTTCTAATTTGGGGATTTTGTGTGACTTGCGCTCTTCAGATCTTGATGAGTCATGCTTGTTGCTTTTTTTCCTCTTTTCTTTCTGCTTTCATGCTTTGATTTCGTGTGCTCACTTGCCTGTACTTCATTTAAAAGGTCTCCACAAAGGGAGACTCAACATTCCCTGCCATTCTGGATAGTTTTGG

**Sequencing results of NSD3-Short Wt.**

CACATGGATTCAGTTTCCAACTGGAGACATGTTACACTTCTGCAAATCCAGGCTCCCTTTGTGCATCGTAATGGAAGCTGGTAAGGA**TTT**CCTTGCTGCCGCAGTTTTCCAGGCTATTTTAACAGGCGGTGGCTCTTCCTCTTCCGCACTTGTGTGCCGCCTCTGGCTATGTCTCCGAATTTCAGTACTTGAGAGTGAGGAGGCCACCTCCCCTGCATTGGTCTGTTCTGGCTGAGTATTCAGCACAGATCTTGGTCGTCGGGTTTTTTTAACTTCGGTTTTGGAGGCAACACTCTTTTTTGCTTGGGATAAAGCCTCTTCAGGCTGTTTATCAATGTAAATAAAAGTATACTGTTCTATTCTTTCTTCTCGAGTCATTTTCAATGCTTTCTCTGCATGGGCAATGCCAATATCCCACTGAGCACGTTCTCTCTGAGGTCGGGGTTTCCGAATCTTTTGTTTCTCAGAGTGATTGCTGGCTTGTTTGGTTGCCTCAGCCAGTAATTCTTCATACTGTTTATGACCTTTATACTCTCGTACCCGTTTTTCATGAACCCACGCCCTCTCTGGCTGGTTGCTAAAAAACTGGACATGATATTCTCGGGCACCTCTTGTGTTAATTTTAGTATGAACCTCAAGCTGGGGATCACTTGAAACCATACAAGGCCACCAAGGATAGGTTCCCACCTTGGACCACACAAGATCGCCAACCTGAAACTTAACACCAGTGGACACTTCCGTTGTTGGAACAGAAGATAGTATTGGCTGAACTGGGGCTTCCTCTTTTAGTACTGGGTTCTTCCCTTGGGTTTTTCTGATACAGTGTCAACCCTCTCATTTGGTCTATTTTGTTCCTCTGGTTCTAATTTGGGGATTTTGTGTGACTTGCGCTCTTCAGATCTTGATGAGTCATGCTTGTTGCTTTTTTTCCTCTTTTCTTTCTGCTTTCATGCTTTGATTTCGTGTGCTCACTTGCCCTGTACTTCATTTAAAAGG

**Sequencing results of K477R Mutant of NSD3-Sort**

CCCATTGGTTTATTTACCACTGGAGACATGTTACACTTCTGCAAATCCAGGCTCCCTTTGTGCATCGTAATGGAAGCTGGTAAGGA**TCT**CCTTGCTGCCGCAGTTTTCCAGGCTATTTTAACAGGCGGTGGCTCTTCCTCTTCCGCACTTGTGTGCCGCCTCTGGCTATGTCTCCGAATTTCAGTACTTGAGAGTGAGGAGGCCACCTCCCCTGCATTGGTCTGTTCTGGCTGAGTATTCAGCACAGATCTTGGTCGTCGGGTTTTTTTAACTTCGGTTTTGGAGGCAACACTCTTTTTTGCTTGGGATAAAGCCTCTTCAGGCTGTTTATCAATGTAAATAAAAGTATACTGTTCTATTCTTTCTTCTCGAGTCATTTTCAATGCTTTCTCTGCATGGGCAATGCCAATATCCCACTGAGCACGTTCTCTCTGAGGTCGGGGTTTCCGAATCTTTTGTTTCTCAGAGTGATTGCTGGCTTGTTTGGTTGCCTCAGCCAGTAATTCTTCATACTGTTTATGACCTTTATACTCTCGTACCCGTTTTTCATGAACCCACGCCCTCTCTGGCTGGTTGCTAAAAAACTGGACATGATATTCTCGGGCACCTCTTGTGTTAATTTTAGTATGAACCTCAAGCTGGGGATCACTTGAAACCATACAAGGCCACCAAGGATAGGTTCCCACCTTGGACCACACAAGATCGCCAACCTGAAACTTAACACCAGTGGACACTTCCGTTGTTGGAACAGAAGATAGTATTGGCTGAACTGGGGCTTCCTCTTTTAGTACTGGTTCTTCCCTTGGTTTTTCTGATACAGTGTCAACCCTCTCATTTGGTCTATTTTGTTCCTCTGGTTCTAATTTGGGGATTTTGTGTGACTTGCGCTCTTCAGATCTTGATGAGTCATGCTTGTTGCTTTTTTTTCCTCTTTTTCTTTTCTGCTTTCATGCTTTGATTTCGTGTGCTCACTTGCCCTGTACTTCATTTAAAAGGTCTCCACAAAGGGAAGACTCAAACATTCCCTGCCATTCTGGATAGTTTGTTA


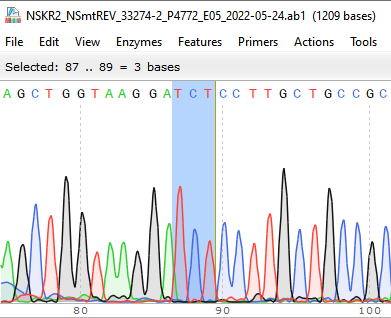


**Arg (R)**

**NSD3S K477R**


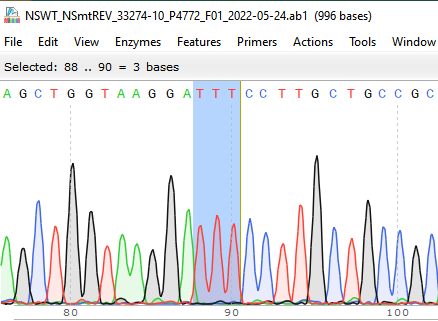


**Lys (K)**

**NSD3S wt**
